# Necroptosis is one of the modalities of cell death accompanying ischemic brain stroke: from pathogenesis to therapeutic possibilities

**DOI:** 10.3325/cmj.2019.60.121

**Published:** 2019-04

**Authors:** Valentina Hribljan, Damir Lisjak, Dražen Juraj Petrović, Dinko Mitrečić

**Affiliations:** Laboratory for Stem Cells, Department for Neurogenetics, Medical Genetics and Regenerative Neuroscience, Croatian Institute for Brain Research, University of Zagreb School of Medicine, Zagreb, Croatia

## Abstract

Due to very limited therapeutic options, ischemic brain injury is one of the leading causes of death and lifelong disability worldwide, which imposes enormous public health burden. One of the main events occurring with ischemic brain stroke is cell death. Necroptosis is a type of cell death described as a regulated necrosis characterized by cell membrane disruption mediated by phosphorylated mixed lineage kinase like protein (MLKL). It can be triggered by activation of death receptors (eg, FAS, TNFR1), which lead to receptor-interacting serine/threonine-protein kinase 3 (RIPK3) activation by RIPK1 in the absence of active caspase-8. Here, we review articles that have reported that necroptosis significantly contributes to negative events occurring with the ischemic brain stroke, and that its inhibition is protective both *in vitro* and *in vivo*. We also review articles describing positive effects obtained by reducing necroptosis, including the reduction of infarct volume and improved functional recovery in animal models. Since necroptosis is characterized by cell content leakage and subsequent inflammation, in addition to reducing cell death, inhibition of necroptosis in ischemic brain stroke also reduces some inflammatory cytokines. By comparing various approaches in inhibition of necroptosis, we analyze the achieved effects from the perspective of controlling necroptosis as a part of future therapeutic interventions in brain ischemia.

Beside cancer and cardiac disease, brain stroke is one of the leading causes of death worldwide. In more than 80% of cases it is caused by blood supply obstruction leading to ischemia (1). Stroke poses an enormous threat to public health, since due to very limited therapeutic options, it frequently leads to a life-long disability or death (1). The period during and after stroke is characterized by the occurrence of metabolic disturbance and inflammatory response, resulting in non-selective death of all cell types. Thus, stroke severity and the course of patient rehabilitation are largely determined by cell death and/or recovery. The occurrence and time frame of reperfusion is also an important factor since restoration of blood and oxygen can induce more robust inflammation and oxidative stress, which can exacerbate ischemic injury. Taking this into consideration, exploring the mechanism of neuronal cell death affected by ischemic stroke is very important for developing efficient clinical treatment (2,3).

Necroptosis is a regulated type of cell death mediated by receptor-interacting serine/threonine-protein kinase 1 (RIPK1), RIPK3, and mixed lineage kinase like protein (MLKL), which results in membrane permeabilization (4). Various animal models have shown that ischemic brain injury is followed by necroptotic cell death, which contributes to its pathology (5-7). In this review, we discuss the effects of inhibition of necroptosis in *in vivo* and *in vitro* models of brain ischemia. We also focus on the role of necroptosis in inflammation following ischemia and the potential role of elements of necroptosis in the nucleus following ischemic brain injury.

## NECROPTOSIS IS A FORM OF REGULATED NECROSIS

Cell death is both a physiological and pathological event present during the development of and in the processes linked to homeostasis. It is also one of the central elements accompanying various pathological processes. For a long time, cell death has been primarily divided into the programmed cell death or apoptosis, and “accidental” cell death or necrosis, which is caused by physiochemical stress. Apoptosis is mediated by proteins, among which are caspases, and is characterized by cytoplasmic shrinkage, chromatin condensation, and plasma membrane blebbing, leading to the formation of small vesicles, which are phagocytosed by neighboring cells (8). On the other hand, necrosis used to be considered as an unregulated form of cell death, which culminates in the cell content leakage, leading to inflammation. Today, we know that cell death of necrotic morphology can be regulated, which means that it can be triggered by a certain mechanism that activates effectors included in the cell membrane disruption. Several forms of regulated necrosis have recently been discovered: necroptosis, ferroptosis, oxytosis, ETosis, NETosis, cyclophilin D-mediated regulated necrosis, parthanatos, and pyroptosis (9). All these types of regulated necrosis are genetically controlled cell death processes that eventually result in cellular leakage and are morphologically characterized by cytoplasmic granulation, as well as organelle and/or cellular swelling (“oncosis”) (9). These forms are involved in different pathological events, and since they are mediated by certain molecules, the inhibition of these molecules represents new therapeutic opportunities for many diseases (10).

**Figure 1:**
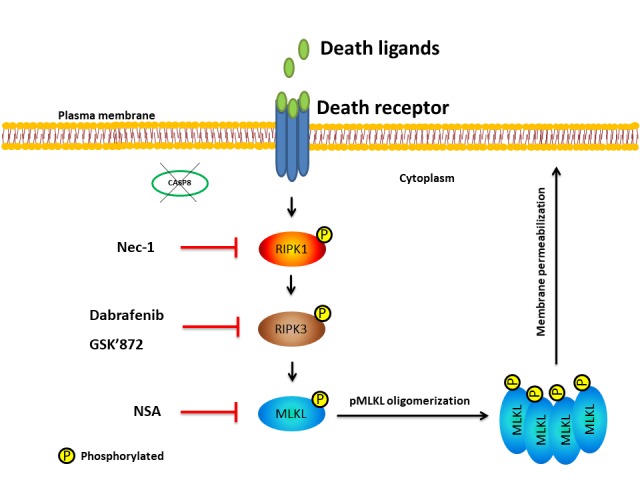
A simplified scheme of necroptotic pathway with inhibitors shown to be protective in *in vitro* and *in vivo* models of ischemic brain stroke. Necroptosis can be triggered by activation of death receptors (eg, FAS, TNFR1), which can lead to receptor-interacting serine/threonine-protein kinase 3 (RIPK3) phosphorylation by receptor-interacting serine/threonine-protein kinase 1 (RIPK1) in the absence of active caspase-8 (CASP8) (11). Phosphorylated RIPK3 binds and phosphorylates mixed lineage kinase like protein (MLKL), resulting in the formation of MLKL oligomers that translocate to the plasma membrane causing its permeabilization (8). The inhibition of necroptosis by necrostatin-1 (Nec-1), dabrafenib, GSK’872, and necrosulfonamide (NSA) has been shown to be protective in *in vitro* and *in vivo* models of ischemic brain stroke.

Necroptosis is the most studied form of regulated necrosis, and its main characteristic is cell membrane disruption mediated by phosphorylated MLKL (pMLKL). Necroptosis can be triggered by the activation of death receptors (eg, FAS, TNFR1), which can lead to RIPK3 activation by RIPK1 in the absence of active caspase-8 (11) (Figure 1). Activated RIPK1 binds RIPK3 through RIP homotypic interaction motif (RIIM) domain in the so called necrosome, in which they undergo reciprocal auto- and trans-phosphorylation (12). Phosphorylated RIPK3 binds and phosphorylates MLKL, thus forming MLKL oligomers, which translocate to the plasma membrane, where they bind to specific phosphatidylinositol phosphate species by a roll-over mechanism and hence trigger plasma membrane permeabilization (8). Since apoptosis and necroptosis can be triggered by the same mechanisms, it is believed that necroptosis has evolved as a “backup” mechanism to ensure cell death when caspases are inhibited (13). For example, during infection with cytomegalovirus, adenovirus, or herpes simplex virus type 1 and 2, apoptosis is prevented by viral inhibition of caspase-8, allowing necroptosis to execute cell death (14). Although activation of necroptosis is beneficial in virus infection, it also contributes to various types of pathological events affecting the cardiovascular system, kidney, liver, and nervous system (15). Taking all this in consideration, it is understandable that blocking necroptosis by RIPK1, RIPK3, or MLKL inhibitors could potentially lead to the development of new drugs for various diseases that include necroptosis as an important negative element.

## INHIBITION OF NECROPTOSIS IS PROTECTIVE IN ISCHEMIC BRAIN INJURY

Necroptosis significantly contributes to negative events occurring with the ischemic brain stroke, and its inhibition is proven to be protective both *in vitro* and *in vivo*. Here, we discuss the effect of inhibition of different elements of necroptosis on infarct volume and neurological/cognitive functions of experimental animals. We focus on specific cell types that are protected by the inhibition of necroptosis following ischemic brain stroke. We also report the effects of inhibition of necroptosis in neurons and astrocytes subjected to oxygen-glucose deprivation (OGD), an *in vitro* model of ischemia. Finally, we describe the effects of inhibition of necroptosis on inflammation following ischemic brain injury and discuss a possible role of elements of necroptosis in the nucleus.

It has been shown that chemical inhibitors of necroptosis are protective in animal models of ischemic brain injury (Table 1) (Figure 1). The first synthesized and the most often used inhibitor of necroptosis is RIPK1-inhibitor necrostatin-1 (Nec-1). The first application of Nec-1 demonstrated its efficiency in inhibition of necroptosis *in vitro*, which was then successfully transferred to a mouse model of ischemic brain injury, where its administration reduced infarct volume (13). Since then, Nec-1 has been used several more times to inhibit necroptosis following stroke, with a confirmed protective effect. Xu et al (16) demonstrated that intracerebroventricular injection of Nec-1 in mice 4 h after reperfusion reduced cerebral infarct volume from 59.3 ± 2.6% to 47.1 ± 1.5% 24 h after middle cerebral artery occlusion (MCAO), measured as a percentage of difference of the affected hemisphere volume to non-affected hemisphere volume. Nec-1 administration has also been shown to be protective in a rat model of MCAO, where its intracerebroventricular injection 30 min before MCAO significantly reduced the infarct volume at 24 h after MCAO (5.08 ± 0.02%) compared with the administration of inactive necrostatin-1 (iNec), after which infarct volume remained high (45.44 ± 0.1%) (6). In addition to reducing lesion size, Nec-1 treatment improved cognitive and neurological functions in animal models of ischemic stroke (6,17).

**Table 1:** The effects of inhibitors of necroptosis in the animal models of ischemic brain stroke*

Marker	Inhibitor	Model of brain ischemia	Effect	Publication
RIPK1	Necrostatin-1	MCAO, mouse	Reduced cerebral infarct volume	(13)
		MCAO, rat	Reduced cerebral infarct volume, improved neurological function	(6)
		MCAO, mouse	Reduced cerebral infarct volume	(16)
		BCAS, mouse	Improved cognitive function	(17)
RIPK3	Dabrafenib	Photothrombosis-induced focal ischemic injury, mouse	Reduced infarct volume	(19)
	GSK’872	MCAO, mouse	Reduced infarct volume	(18)
MLKL	Necrosulfonamide	MCAO, mouse	Reduced cerebral infarct volume, improved neurological function	(20)

*RIPK – receptor-interacting serine/threonine-protein kinase; MLKL – mixed lineage kinase like protein; MCAO – middle cerebral artery occlusion; BCAS – bilateral common carotid artery stenosis.

Inhibition of RIPK3 and MLKL, downstream regulators of necroptosis, has also been shown to be protective in ischemic brain stroke. The administration of RIPK3 inhibitors, GSK’872 or dabrafenib, reduced lesion size in mice subjected to ischemic brain injury (18,19). In addition, the intracerebroventricular administration of MLKL inhibitor necrosulfonamide (NSA) 30 min before MCAO improved neurological functions of mice and reduced infarct volume (20). The intracerebral injection of NSA 4 h after MCAO also significantly reduced infarct volume, while administration of NSA 6 h post reperfusion did not show a neuroprotective effect on infarct volume, suggesting that NSA had a therapeutic window after ischemic injury (20).

The protective effect of inhibition of necroptosis, seen as a reduction of infarct volume and improvement in neurological functions, is primarily achieved by the protection of the brain cells. Ni et al (5) demonstrated that at 12 h of permanent MCAO (pMCAO), mice's cortical neurons and astrocytes showed necrotic morphological features, but Nec-1 treatment improved their morphology and reduced necrotic death of both cell types. They also showed that short hairpin RNA RIPK1 or Nec-1 treatment prevented pMCAO and decreased MAP2 or GFAP levels in the ischemic cortex 12 h after pMCAO. Inhibition of necroptosis significantly improved the survival of hippocampal CA1 neurons: morphologically, only 9.3 ± 1.3% and 7.4 ± 3.0% of the hippocampal CA1 neurons in the control group survived at 7 and 30 days, respectively, as opposed to 90.9 ± 1.1% and 86.7 ± 1.3%, respectively, in the Nec-1 group. This strongly suggests that Nec-1 has a long-term protective effect against hippocampal CA1 neuronal death rather than just delaying death (21). Although necroptosis is involved in axonal degeneration during the progress of neurodegenerative diseases (22), to the best of our knowledge its involvement in axonal degeneration in ischemic brain damage has not been shown.

Inhibition of necroptosis in neurons subjected to OGD has also been shown to have a protective role. Treatment of rat cortical neurons with Nec-1 before OGD significantly reduced the percentage of necrotic cell death measured 3 h post recovery: in OGD group the percentage of necrotic cell death was 60.5 ± 5.8%, while in the OGD+Nec1 group it was 40.8 ± 4.3% (23). Pretreatment of mice's cortical neurons with Nec-1 increased cell viability after OGD-induced damage from 47.9 ± 1.1% in controls without treatment to 68.4 ± 1.6% (16). Furthermore, Nec-1 or short hairpin RNA RIPK1 treatment not only protected neurons, but also reduced cell death of astrocytes subjected to OGD (5). Finally, treatment of HT 22 cells (a mouse hippocampal cell line) 14 h after OGD by GSK’872 increased their number 9 h after oxygenation compared with untreated control (18).

In our own experiments, we analyzed how neural stem cell transplantation after stroke influenced the expression of necroptosis markers. Interestingly, we found that neural stem cell transplantation, but also enriched medium, significantly down-regulated *Ripk*1. At the same time, neural stem cells did not influence *Ripk3* and *Mlkl* levels, which remains to be explained (24).

Inhibitors specific for a certain element of necroptosis affect not only an isolated path but also the whole necroptosis pathway. MLKL small interfering RNA notably inhibited MLKL expression and RIPK1-RIPK3-MLKL interaction in the rat cortex and hippocampus subjected to hypoxia-ischemia (25). Ni et al showed that Nec-1 treatment inhibited pMCAO- or OGD-induced increase in the interaction between RIPK1 and RIPK3 (5). GSK’872 treatment decreased RIPK1, RIPK3, and MLKL expression and inhibited the phosphorylation of MLKL compared with the non-treated group *in vitro* and *in vivo* (18). Nec-1 treatment in a model of bilateral common carotid artery stenosis (BACS) not only decreased the expression of RIPK1 but also that of RIPK3 (17).

Since necroptosis is characterized by cell membrane rupture and cell content leakage, one of the most obvious effects of inhibition of necroptosis is the reduction of inflammation. Inhibition of necroptosis by Nec-1 following BACS has been shown to have long term effects manifested in the down-regulation of inflammatory cytokines TNF-α, INF-γ, IL-1β, and IL-33, 15 days after treatment both on mRNA and protein levels (17). Although Nec-1 is a specific RIPK1 inhibitor (26), its administration 30 min before MCAO, in addition to suppression of RIPK3, MLKL, and phosphorylated MLKL, also suppressed the generation of mature IL-1β – one of the most important inflammatory cytokines in the rat brain (6). By conducting *in vitro* experiments using conditioned medium generated from WT and RIPK3^−/−^ neurons to treat macrophages, Yang et al (27) showed that necroptotic neurons probably facilitated inflammation through the induction of the M1 fate of microglia/macrophages, while necroptosis-defective neurons may favor M2 polarization by releasing distinct inflammatory factors.

Necroptosis is a specific type of cell death mediated by RIPK1, RIPK3 and MLKL, resulting in cell membrane permeabilization by MLKL (Figure 1). On the other hand, the role of elements of necroptosis in the nucleus is less clear. It has been long thought that one of the main differences between apoptosis and cell death of necrotic morphology is the presence of DNA fragmentation in the first form of cell death, and its absence in the latter, but recently published articles question this concept. It seems that the association of MLKL with the cell membrane in necroptotic death is preceded by the translocation of pMLKL, along with RIPK1 and RIPK3, to the nucleus, and translocation of pMLKL to the nucleus is probably not required for necroptosis but might facilitate it (28). Furthermore, Weber et al (29) suggested that the passage of necroptotic signaling components through the nucleus was a mechanism for regulating cytosolic necrosome formation. Regarding ischemic brain injury, it has been shown that in the striatum of ischemic stroke rats 24 h post MCAO, most of pRIPK1 positive cells exhibited DNA double-strand breaks (6). In support of the thesis that necroptosis may be involved in DNA fragmentation, Xu et al (21) suggested that after ischemia-reperfusion injury in hippocampal CA1 neurons, RIPK3 forms a complex with AIF, which then translocates to the nucleus. This results in chromatin condensation and DNA degradation, a process which may be inhibited by pre-treatment with Nec-1 (21).

## CONCLUSION

Necroptosis has become one of the most studied forms of cell death because of its presence in many pathological events. Recent articles clearly showed that necroptosis is responsible for a large portion of negative effects occurring after brain ischemia. Equally important, inhibition of elements of necroptosis significantly reduces brain damage. Taken altogether, necroptosis entered the scenery of brain pathophysiology as one of its central elements. Current efforts focused on better understanding how to control necroptosis have a potential to lead to new therapeutic options for reducing damage caused by brain ischemia.

## References

[1] Flynn RWV, Macwalter RSM, Doney ASF (2008). The cost of cerebral ischaemia.. Neuropharmacology.

[2] Pundik S, Xu K, Sundararajan S (2012). Reperfusion brain injury Focus on cellular bioenergetics.. Neurology.

[3] Li P, Shen M, Gao F, Wu J, Zhang J, Teng F (2017). An Antagomir to MicroRNA-106b-5p Ameliorates Cerebral Ischemia and Reperfusion Injury in Rats Via Inhibiting Apoptosis and Oxidative Stress.. Mol Neurobiol.

[4] Grootjans S, Vanden Berghe T, Vandenabeele P (2017). Initiation and execution mechanisms of necroptosis: an overview.. Cell Death Differ.

[5] Ni Y, Gu WW, Liu ZH, Zhu Y, Rong J, Kent TA (2018). RIP1K contributes to neuronal and astrocytic cell death in ischemic stroke via activating autophagic-lysosomal pathway.. Neuroscience.

[6] Deng X, Li S, Sun F (2018). Necrostatin-1 Prevents Necroptosis in Brains after Ischemic Stroke via Inhibition of RIPK1-Mediated RIPK3 / MLKL Signaling.. Aging Dis.

[7] Zhang S, Wang Y, Xie H, Yu Q, Wu J, Wu Y (2017). Necroptosis and microglia activation after chronic ischemic brain injury in mice.. Eur J Inflamm.

[8] Galluzzi L, Vitale I (2018). Molecular mechanisms of cell death: recommendations of the Nomenclature Committee on Cell Death 2018.. Cell Death Differ.

[9] Vanden Berghe T, Linkermann A, Jouan-Lanhouet S, Walczak H, Vandenabeele P (2014). Regulated necrosis: the expanding network of non-apoptotic cell death pathways.. Nat Rev Mol Cell Biol.

[10] Conrad M, Angeli JPF, Vandenabeele P, Stockwell BR (2016). Regulated necrosis: disease relevance and therapeutic opportunities.. Nat Rev Drug Discov.

[11] Vanden Berghe T, Kaiser WJ, Bertrand MJ, Vandenabeele P (2015). Molecular crosstalk between apoptosis, necroptosis, and survival signaling.. Mol Cell Oncol.

[12] Vandenabeele P, Declercq W, Van Herreweghe F, Vanden Berghe T, Vanden Berghe T (2010). The role of the kinases RIP1 and RIP3 in TNF-induced necrosis.. Sci Signal.

[13] Degterev A, Huang Z, Boyce M, Li Y, Jagtap P, Mizushima N (2005). Chemical inhibitor of nonapoptotic cell death with therapeutic potential for ischemic brain injury.. Nat Chem Biol.

[14] Mocarski ES, Upton JW, Kaiser WJ (2011). Viral infection and the evolution of caspase 8-regulated apoptotic and necrotic death pathways.. Nat Rev Immunol.

[15] Galluzzi L, Kepp O, Chan FK-M, Kroemer G (2017). Necroptosis: Mechanisms and Relevance to Disease.. Annu Rev Pathol.

[16] Xu X, Chua K-W, Chua CC, Liu C-F, Hamdy RC, Chua BHL (2010). Synergistic protective effects of humanin and necrostatin-1 on hypoxia and ischemia/reperfusion injury.. Brain Res.

[17] Zhang S, Wang Y, Li D, Wu J, Si W, Wu Y (2016). Necrostatin-1 Attenuates Inflammatory Response and Improves Cognitive Function in Chronic Ischemic Stroke Mice.. Medicines (Basel).

[18] Yang X-S, Yi T-L, Zhang S, Xu Z-W, Yu Z-Q, Sun H-T (2017). Hypoxia-inducible factor-1 alpha is involved in RIP-induced necroptosis caused by in vitro and in vivo ischemic brain injury.. Sci Rep.

[19] Cruz SA, Qin Z, Stewart AFRR, Chen H-HH (2018). Dabrafenib, an inhibitor of RIP3 kinase dependent necroptosis, reduces ischemic brain injury.. Neural Regen Res.

[20] Zhou Y, Zhou B, Tu H, Tang Y, Xu C, Chen Y (2017). The degradation of mixed lineage kinase domain-like protein promotes neuroprotection after ischemic brain injury.. Oncotarget.

[21] Xu Y, Wang J, Song X, Qu L, Wei R, He F (2016). RIP3 induces ischemic neuronal DNA degradation and programmed necrosis in rat via AIF.. Sci Rep.

[22] Ito Y, Ofengeim D, Najafov A, Das S, Saberi S, Li Y (2016). RIPK1 mediates axonal degeneration by promoting inflammation and necroptosis in ALS.. Science.

[23] Yuan L, Wang Z, Liu L, Jian X (2015). Inhibiting histone deacetylase 6 partly protects cultured rat cortical neurons from oxygen-glucose deprivation-induced necroptosis.. Mol Med Rep.

[24] Hribljan V, Salamon I, Đemaili A, Alić I, Mitrečić D (2018). Transplantation of neural stem cells in the mouse model of ischemic brain stroke and expression of genes involved in programmed cell death.. Croat Med J.

[25] Qu Y, Shi J, Tang Y, Zhao F, Li S, Meng J (2016). MLKL inhibition attenuates hypoxia-ischemia induced neuronal damage in developing brain.. Exp Neurol.

[26] Degterev A, Hitomi J, Germscheid M, Ch’en IL, Korkina O, Teng X (2008). Identification of RIP1 kinase as a specific cellular target of necrostatins.. Nat Chem Biol.

[27] Yang J, Zhao Y, Zhang L, Fan H, Qi C, Zhang K (2018). RIPK3/MLKL-mediated neuronal necroptosis modulates the m1/m2 polarization of microglia/macrophages in the ischemic cortex. Cereb Cortex.

[28] Yoon S, Bogdanov K, Kovalenko A, Wallach D (2016). Necroptosis is preceded by nuclear translocation of the signaling proteins that induce it.. Cell Death Differ.

[29] Weber K, Roelandt R, Bruggeman I, Estornes Y, Vandenabeele P (2018). Nuclear RIPK3 and MLKL contribute to cytosolic necrosome formation and necroptosis.. Commun Biol..

